# Urban–Rural Differences in Subjective Well-Being of Older Adult Learners in China

**DOI:** 10.3389/fpsyg.2022.901969

**Published:** 2022-07-14

**Authors:** Xu Jiayue, Ye Changsheng, Sun Lixin, Yu Xiao, Zhao Wenjun, Le Chuanyong

**Affiliations:** ^1^College of Teacher Education, Ningbo University, Ningbo, China; ^2^Institute of Elderly Education, Ningbo Open University, Ningbo, China

**Keywords:** subjective well-being, urban–rural differences, older adults, empirical analysis, older adult education

## Abstract

Population aging has brought great challenges to many regions throughout the world. Enhancing the sense of participation, access, and well-being of older adults is the goal of China’s aging development. This study, taking urban–rural difference as the entry point, examined the difference in subjective well-being between urban and rural older learners. A total of 2,007 older adults learners (*n* = 2007) aged over 50 years were recruited in Zhejiang, Anhui, and Shandong Provinces in China, including 773 rural older adults and 1,234 urban older adults. This study found that there was a significant positive correlation between senior learning and the subjective well-being of urban and rural older adult learners. Furthermore, there was a significant difference between the subjective well-being of urban and rural older adult learners’ and there was also an urban–rural difference between the effects of older adult learning on the subjective well-being. Based on the above findings, this study reveals the mechanism of the impact of older adult learning on subjective well-being of urban and rural older adults and gives relevant suggestions for improving the subjective well-being of urban and rural older learners.

## Introduction

With economic development and social progress, the degree of national attention to public well-being and access will become critical to the quality of working life and social development. Researchers generally agree that subjective well-being is an important criterion for measuring whether a country or region has achieved successful aging (Tovel and Carmel, 2014). Population aging is increasing with an irresistible trend. The Chinese seventh national census [National Bureau of Statistics of China (NBSC), 2020] shows that China’s population aged 60 and over was 264.02 million, accounting for 18.70% of the population, and the population aged 65 and over was 190.64 million, accounting for 13.5%. Enhancing the happiness of the older adults is not only related to the quality of life of more than 200 million older adults, but also related to the working life of their children and the harmony and stability of society (Wang, 2021). China attaches great importance to meeting the spiritual needs of the elderly, enabling them to share the fruits of development and enjoy a happy old age (The State Council, 2016, 2022). Focus on older adults’ subjective well-being (SWB) is therefore highly relevant.

Researchers pointed out that participation in educational activities is helpful for improving SWB; however, because unbalanced development between urban and rural areas is an important characteristic of China’s economic and social development, the education disparity is an important manifestation of it (Zhang, 2017). Older adult education in rural areas is lacking in funding, hardware and software infrastructure, educational institutions, and teachers and faculty (Lu, 2020). The Chinese government is committed to narrowing the development gap between urban and rural areas, achieving integrated urban and rural development, equalizing public services, and building a model lifelong learning society (The State Council, 2021). In light of the disparity, it is important to investigate the current situation of the SWB of older adult learners in urban and rural areas, which plays a significant role in the equalization of the elderly education. Previous studies have mainly focused on the influence of objective factors on older adults SWB, such as social support, the Internet, and socioeconomic status (Choi et al., 2021; Deng, 2021; Wang, 2021; Ye and Li, 2022); few studies have analyzed the differences between older adults’ SWB from the perspective of older adult education. Therefore, this paper intends to investigate the differences between the SWB of older learners in urban and rural areas and the influencing factors and provide a theoretical basis for promoting equity in older adult education and enhancing of older adults’ SWB.

### SWB of Older Adults

Research on SWB has used various orientations and measurement indicators. In terms of orientation, the marginal utility theory founded by Jevons initiated the research from an economic perspective (Sun, 2004). Since then, other scholars have conducted research from a psychological perspective, focusing more on individuals’ SWB, which comprises quality of life, mental health, and psychological development (Xing, 2002).

In terms of indicators for measuring SWB, psychological development indicators have been added beyond the initial indicators of quality of life and mental health (Li, 2006), including strength, sense of autonomy, pleasure, self-confidence, and others. Self-report scales and timely measurement methods have gradually been developed (Yu, 2018). The former mainly includes the Satisfaction with Life Scale, the General Well-Being Schedule, the Memorial University of Newfoundland Scale of Happiness (MUNSH), and the Multidimensional Psychological Well-being Scale. The latter mainly includes the day reconstruction method, ecological momentary assessment, and experience sampling method. Among these, the Philadelphia Geriatric Center Morale Scale and the MUNSH have most commonly been used to measure older adults’ SWB.

Research on SWB in China began in the mid-1980s, and the findings mainly involved economics, psychology, and sociology. The focus has since shifted from qualitative description to quantitative measurement. In the early research stage, domestic scholars mostly borrowed research ideas and measurement tools from foreign scholars; for example, Yang et al. (1988) used the MUNSH to study the happiness of older adults. With the depth of domestic research, the development of localized measurement tools has received increasing attention and many scholars have revised international SWB scales accordingly based on practical needs.

Xing and Liu (2008) proposed the idea of experiential SWB with both form and content, and his Subjective Well-Being Scale for Chinese Citizens (SWBS-CC) has been well accepted in China. The scale contains 10 dimensions of interpersonal adaptation experience, mental health experience, family atmosphere experience, and so on, which reveal the SWB of Chinese citizens (including older adults) in a more comprehensive way. In this study, the SWBS-CC was adjusted based on actual survey data to measure the SWB of older learners in three dimensions: adaptation satisfaction experience, physical and mental health experience, and self-development experience (Sun and Liu, 2020).

### The Relationship Between Older Adult Learning and SWB

Studies have reached different conclusions regarding the relationship between learning activities and SWB. Some scholars believe that there is a positive correlation between learning and SWB; the higher the level of education, the higher the individual’s SWB (Yue et al., 2021). Researchers have noted that education level is an important factor influencing the overall life satisfaction of older adults (Cheng and Yan, 2021). Older adults’ motivation to participate in lifelong education plays an important role in learning satisfaction, which has a positive impact on quality of life (Kim and Lim, 2014; Hu, 2019), and teachers and fellow students have the greatest influence on the quality of life of older learners (Escuder-Mollon et al., 2014). Meanwhile, participation in learning activities can promote quality of life in older adults, effectively resolve their inner conflicts, and help improve their SWB (Bolton-Lewis and Li, 2017; Sun and Liu, 2020; Xiang and Li, 2020; Feng and Liu, 2022). However, some scholars have also found that the correlation between participation in learning activities and SWB is not necessarily significant or can even be negative (Powdthavee et al., 2015) because the premise that adults’ participation in lifelong learning activities positively affects their mental health and quality of life is consistent with their learning interests (Hammond, 2004).

### Differences in SWB of Urban and Rural Older Adult Learners and Their Influencing Factors

At present, it is common to study SWB in entire populations, and few studies have specifically examined older adult learners. Some scholars believe that there is a significant difference between the SWB of urban and rural residents, and urban residents are happier than rural residents (Zhou, 2015). In contrast, Luo (2006) came to the opposite conclusion by using explicit variables to measure happiness. Su and Zhang (2021) concluded that rural residents’ social class and income had a significant effect on their SWB; however, although older adults with agricultural household registration were generally less satisfied with their lives than those with non-agricultural household registration, they scored higher in positive affect, positive experience, and overall happiness in rural areas than in urban areas (Jiang and Lin, 2008). Besides, the expansion of unequal opportunities will reduce rural adults’ SWB (Yu and Zhang, 2019; Zhang and Wan, 2020).

Most research on the factors influencing the SWB of older adult learners has involved demographic and psychological variables, such as age, gender, education, aging attitudes, and self-perceptions. Research from the perspective of learning participation has been conducted in terms of learning motivation, learning needs, learning intentions, and learning opportunities. Some researchers have identified the factors in terms of their engagement in learning activities, learning atmosphere, and learning experience (Sun and Liu, 2020; Sun and Zhang, 2021). Fan (2009) pointed out that older adults’ participation in learning activities includes six factors that can greatly enhance their SWB: learning atmosphere, learning interest, learning initiative, learning difficulties and challenges, and learning commitment, and participation (Bo, 2017). Several studies found that the creation of “learning circles” and “social circles” can realize the self-worth of older participants and promote interpersonal communication, thus affecting the quality of older adults’ happiness (Le and Xia, 2016; Gu and Wang, 2020).

Previous studies on older adult learning and SWB have mainly focused on the study of education level’s influence of on older adult learners’ SWB. Few scholars have explored the difference in learning participation’s influence on urban and rural older adult learners’ SWB (Xiang and Yao, 2016), which is the concern of this study. Based on the above research, the current study sought to investigate whether there are differences in older adult learning’s influence on urban and rural older adults’ SWB and the factors contributing to that difference. The specific hypotheses were as follows:

*Hypothesis 1*: There is a significant positive relationship between older adult learning and the SWB of urban and rural older learners.
*Hypothesis 2*: There is a significant difference between the SWB of urban and rural older adult learners.
*Hypothesis 3*: The positive effect of older adult learning on the SWB of rural older adults is higher than that of urban older adults.

## Materials and Methods

### Participants and Procedures

**Figure 1:**
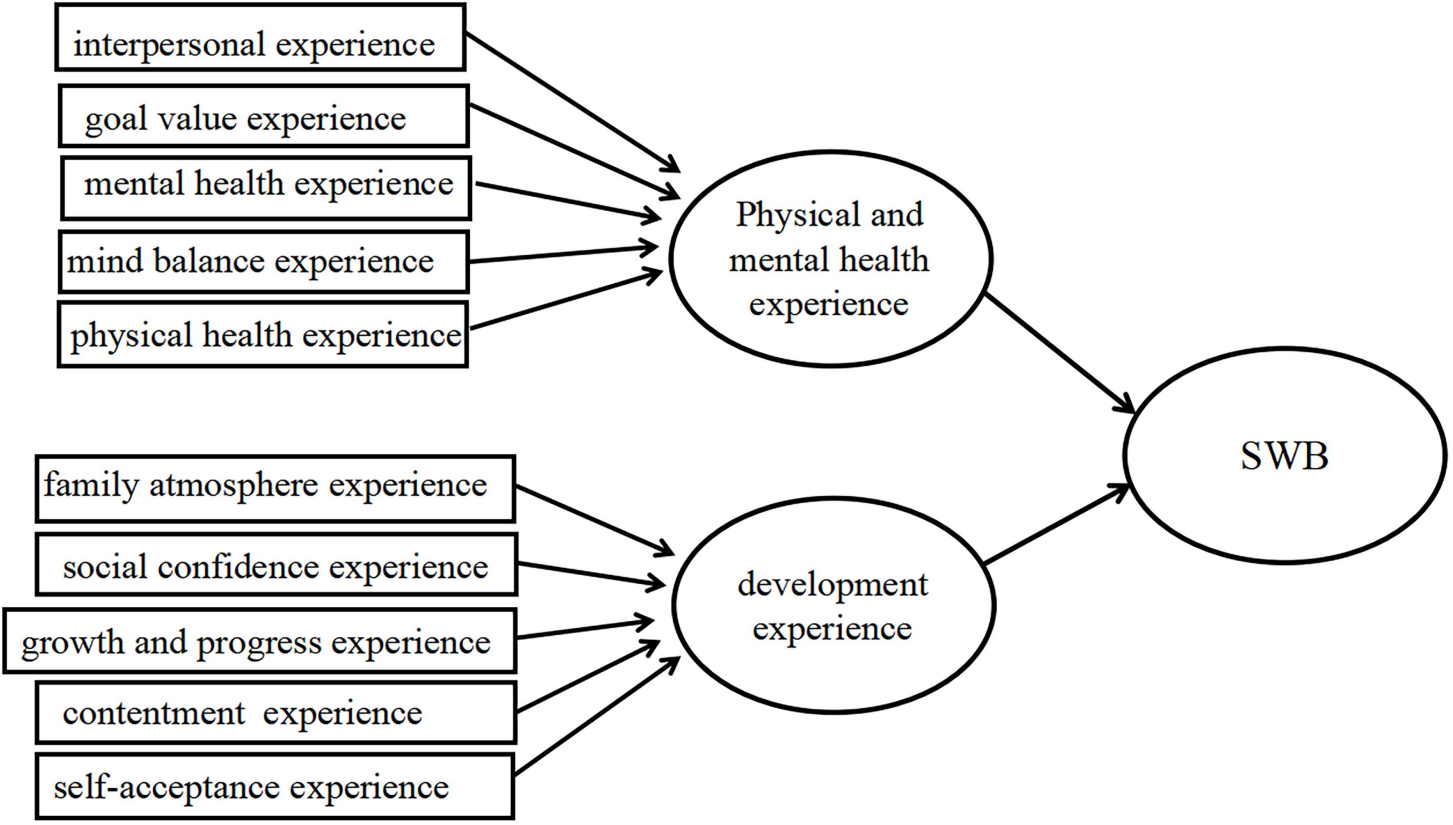
The model of modified Subjective Well-Being Scale for Chinese Citizens (SWBS-CC).

The modified SWBS-CC (See Figure 1; Xing and Liu, 2008) was used to obtain survey data from the middle and eastern regions of Zhejiang, Anhui, and Shandong Provinces, where senior education is better developed and the representation is more prominent. A random sampling method was adopted to randomly distribute electronic questionnaires to urban and rural residents over 50 years old (defined by the enrollment requirements of senior colleges) who participate in learning through educational institutions, such as senior colleges, community colleges, and adult schools (based on household registration location). At the beginning of the study, the inclusion criteria and the study goals were explained to all participants, and their consent to participate was obtained. For the participants who could not read or understand questionnaires independently, the investigators read questions one by one helped them fill in the questionnaires. Participants were assured that confidentiality and anonymity would be maintained. Ultimately, 2,007 valid questionnaires were obtained, with a valid questionnaire recovery rate of over 85%. The final samples consisted of 1,234 urban older adult learners (61.5%) and 773 rural older adult learners (38.5%). Table 1 provides the demographic characteristics of the participants in the current study.

**Table 1:** Participants’ demographic characteristics.

	Total	Rural	Urban
Total individuals	2,007	773	1,234
**Age**			
50–59	948 (47.2%)	464 (48.9%)	484 (51.1%)
60–69	785 (39.1%)	229 (29.2%)	556 (70.8%)
70–79	262 (13.1%)	77 (29.4%)	185 (70.6%)
over 80	12 (0.6%)	3 (25.0%)	9 (75.0%)
**Gender**			
Male	264 (13.2%)	95 (36.0%)	169 (64.0%)
Female	1743 (86.8%)	678 (38.9%)	1,065 (61.1%)
**Marital status**			
Single	180 (9%)	59 (32.8%)	121 (67.2%)
Married	1827 (91%)	714 (39.1%)	1,113 (60.9%)
**Monthly income**			
Under ¥1,000	95 (4.7%)	55 (57.9%)	40 (42.1%)
¥1,000–¥2,000	500 (24.9%)	335 (67.0%)	165 (33.0%)
¥2,000–¥4,000	785 (39.1%)	250 (31.8%)	535 (68.2%)
Over ¥4,000	627 (31.2%)	133 (21.2%)	494 (78.8%)
**Education level**			
Less than junior middle school	177 (8.8%)	125 (70.6%)	52 (29.4%)
Junior middle school	836 (41.7%)	421 (50.4%)	415 (49.6%)
High school (include technical secondary school and professional high school)	697 (34.7%)	193 (27.7%)	504 (72.3%)
Junior college	203 (10.1%)	23 (11.3%)	180 (88.7%)
College/Bachelor’s degree or above	94 (4.7%)	11 (11.7%)	83 (88.3%)

### Study Measures

#### Demographic Variables

The questionnaire used had three parts. The first asked about demographic variables in two sections: family characteristics, such as marital status and whether they live alone, and individual characteristics, such as gender, age, education, and income.

#### Older Adult Learning

Older adult learning was the independent variable in this study. In this scale, exploratory factor analysis (EFA) was first used to identify the factors included in older adult learning. The objective of this analysis was to establish the variables to be introduced in the confirmatory factor analysis (CFA) model. The EFA identified three factors: learning investment, learning atmosphere, and learning experience. The measurement of learning investment refers to the measurement method of family economic status (Jin et al., 2017), which mainly involves three items: the period of learning, the number of courses, and the cost of education. Among them, the period of learning assignment method is 1 (less than 1 year) to 4 (more than 10 years), the number of courses is assigned as 1 (one) to 4 (more than four), and the cost of education assignment method is 1 (0 yuan) to 5 (more than 200 yuan per month). The indicators of learning investment are summed after the above variables are standardized.

The learning atmosphere and learning experience act as both independent and intermediate variables. Learning atmosphere was mainly measured from three directions: teachers’ teaching ability, teacher–student relationship, and the relationship between students, which is composed of three items (e.g., “The teacher’s lectures were very lively and interesting, and I relished them every time.”), using a six-point scale from 1 (strongly disagree) to 6 (strongly agree); the higher the score, the better the educational atmosphere. Learning experience was mainly measured from the directions of learning interest and learning initiative, challenge difficulty and the importance of learning, and so on, composed of five items (e.g., “Whether it’s attending a class or a club activity, I’m always highly focused.”), using a six-point scale to assign a value of 1 (strongly disagree) to 6 (strongly agree). The higher the score, the better the educational experience is. The Cronbach’s alpha value for the total scale was 0.858 (*α* = 0.858), and the CFA model had an acceptable statistical fit, according to Brown (2006) (=*x*^2^/*df* = 1.762, CFI = 0.99, TLI = 0.986, GFI = 0.972, RMSEA = 0.041). Tables 2, 3 indicate that the composite reliability of variables is higher than 0.6, the Average Variance Extracted (AVE) of learning atmosphere and learning experience were higher than 0.5 and the AVE of learning investment was higher than 0.4. Fornell and Larcker (1981) said that if AVE is less than 0.5, but composite reliability is higher than 0.6, the convergent validity of the construct is still adequate; the square root of the convergent validity and the coefficients are greater than the intercorrelations among the factors, which shows acceptable reliability and validity.

**Table 2:** Results of confirmatory factor analysis (CFA) for older adult learning.

Variables	Items	Estimate	AVE	CR
Learning investment	Number of courses	0.6		
	Cost of education	0.655		
	Period of learning	0.691	0.422	0.686
	The relationship between learners	0.888		
	The relationship between learners and teachers	0.873	0.7623	0.9058
Learning atmosphere	Teachers’ teaching ability	0.858		
	Learning concentration	0.879		
	Learning interest	0.781	0.7186	0.9272
	Learning initiative	0.832		
	Challenge difficulty	0.852		
Learning experience	The importance of learning	0.89		

**Table 3:** Correlation between older adult learning subscales.

	Learning investment	Learning atmosphere	Learning experience
Learning investment	**0.422**		
Learning atmosphere	0.214	**0.7623**	
Learning experience	0.264	0.478	**0.7186**
Square			
root of the AVE	0.650	0.873	0.848

The bold values are the values of AVE.

#### Subjective Well-Being

The third part of the questionnaire was the dependent variable of SWB, which included three measurement dimensions: physical and mental health experience, adaptation satisfaction experience, and self-development experience. The research scale was based on the SWBS-CC (Xing and Liu, 2008) and was adjusted according to the research needs. The experience of adaptation and satisfaction is measured from the experience of family atmosphere, interpersonal adaptation, contentment, and social confidence (four items, e.g., “Since attending the Senior University, I have become more and more willing to participate in various group activities and make many new friends.”). The physical and mental health experience is measured from the experience of physical health, mental health, and mental balance (three items, e.g., “Since participating in older adults’ education activities, I have not felt very annoyed whenever my physical health is not good.”). The self-development experience is measured from the experience of growth and progress, target value, and self-acceptance (three items, e.g., “Since participating in the education activities for older adults, I feel that I have become stronger, more capable, and more able to keep up with the development of society.”). The scale consists of 10 items rated on a six-point scale, ranging from 1(strongly disagree) to 6 (strongly agree) with higher scores indicating higher SWB of older learners. The Cronbach’s alpha values for physical and mental health experience, adaptation satisfaction experience, and self-development experience are 0.915, 0.915, and 0.933, respectively. The CFA model has an acceptable statistical fit, according to Brown (2006) (=*x*^2^/*df* = 1.157, CFI = 0.984, TLI = 0.997, GFI = 0.984, RMSEA = 0.019). Tables 4, 5 show that the composite reliability of variables is higher than 0.7, the AVE of three dimensions of SWB is higher than 0.5, and the square root of the convergent validity and the coefficients are greater than the intercorrelations among the factors, indicating that validity and reliability of the scale are good and meet the research requirements.

**Table 4:** Results of CFA for SWB.

Variables	Items	Estimate	AVE	CR
The physical and mental health experience	Physical health	0.838	0.671	0.8594
	Mental health	0.808		
	Mental balance	0.811		
The self-development experience	Self-acceptance	0.808	0.7164	0.8833
	Target value	0.869		
	Growth and progress	0.861		
The experience of adaptation and satisfaction	Family atmosphere	0.848	0.6871	0.8978

The bold values are the values of AVE. The square square root of the convergent validity and the coefficients is greater than the intercorrelations among the factors, which shows a great discriminant validity

**Table 5:** Correlation between SWB subscales.

	The physical and mental health experience	The self-development experience	The experience of adaptation and satisfaction
The physical and mental health experience	**0.671**		
The self-development experience	0.56	**0.716**	
The experience of adaptation and satisfaction	0.58	0.565	**0.6871**
Square root of the AVE	0.819	0.846	0.829

The bold values are the values of AVE.

### Data Analyses

We used SPSS 21.0 as the research tool for this study. First, descriptive statistical analysis and difference test were used to analyze the differences in the SWB of older adult learners and the dimensions of older adult learning in terms of demographic variables. Second, correlation analysis was used to verify whether older adult learning (learning investment, learning experience, and learning atmosphere) affects the SWB of urban and rural older adult learners. Independent *t*-test was used to verify whether there was a significant difference in SWB between urban and rural older learners. Finally, multiple linear regression was used to examine the mechanism of older adult learning on the SWB of urban and rural older learners and to discover the differences in the impact effect of older adult learning on enhancing the SWB of urban and rural older adults.

## Results

### Descriptive Statistical Analysis

Means, SDs, and possible ranges were calculated for main study variables in the current study. According to the research data (Table 6), there were significant gender differences in SWB, with women significantly higher than men (5.55 ± 0.757 vs. 5.25 ± 0.955, *t* = −5.716, *p* < 0.01); there were significant age differences in participants’ SWB, with those aged 50–59 having the highest SWB and those aged over 80 having the lowest (*F* = 15.217, *p* < 0.01); there were also significant differences in SWB of participants with different incomes (*F* = 4.858, *p* < 0.01); SWB was highest for participants with disposable income between RMB 1,000 and 2,000. There were also significant differences in SWB related to education level; specifically, the lower the education level, the higher the SWB (*F* = 15.761, *p* < 0.01).

**Table 6:** Descriptive Analysis.

	SWB	Educational atmosphere	Educational experience	Educational investment
Male	5.25±0.955	5.27±0.885	5,27±0.959	3.80±0.662
Female	5.55±0.756	5.55±0.731	5.55±0.736	3.80±0.637
*t*	−5.716^***^	−5.502^***^	−5.450^***^	−1.97
Married	5.52±0.78	5.52±0.744	4.07±1.17	3.8±0.634
Single	5.44±0.899	5.45±0.89	3.93±1.06	3.87±0.696
*t*	1.205^*^	−0.89^*^	0.748^*^	−1.488
50–59	5.55±0.737	5.56±0.682	5.56±0.707	3.7±0.582
60–69	5.51±0.795	5.51±0.769	5.52±0.775	3.85±0.653
70–79	5.39±8.878	5.39±0.886	5.37±0.894	4.04±0.708
Over 80	4.14±1.247	4.28±1.355	4.07±1.26	3.5±0.823
*F*	15.217^***^	14.723^***^	18.439^***^	22.892^***^
Under ¥1000	5.45±0.956	5.41±1.028	5.41±0.999	3.45±0.666
¥1000–¥2000	5.62±0.704	5.58±0.737	5.61±0.69	3.65±0.616
¥2000–¥4000	5.5±0.788	5.51±0.73	5.52±0.761	3.78±0.628
Over ¥4000	5.44±0.827	5.46±0.76	5.44±0.807	4.01±0.607
*F*	4.858^*^	2.844^*^	5.083^*^	43.526^***^
Less than junior middle school	5.67±0.745	5.63±0.792	5.63±0.799	3.61±0.691
Junior middle school	5.59±0.721	5.58±0.693	5.59±0.694	3.72±0.644
High school (include technical secondary school and professional high school)	5.5±0.758	5.5±0.749	5.51±0.752	3.89±0.614
Junior college	5.25±0.939	5.27±0.87	5.23±0.919	3.96±0.626
College/bachelor’s degree or above	5.1±1.077	5.25±0.893	5.2±0.996	3.94±0.529
*F*	15.761^***^	11.107^***^	13.958^***^	14.777^***^

* *p* < 0.05;

^**^p < 0.01;

*** *p* < 0.001.

There were significant gender differences in learning atmosphere and learning experience in the dimensions of older adult learning, with females scoring higher than males; the learning investment, learning atmosphere, and learning experience of older seniors were significantly higher than those of younger seniors. There were also significant differences in learning investment, learning atmosphere, and learning experience among participants with different incomes and education levels; specifically, older adults with incomes above RMB 4,000 had the highest learning commitment, and those with lower education levels had the highest learning experience. The learning investment and learning atmosphere of older adults with lower education levels were better.

### Correlation Analysis of Senior Learning and SWB

To identify the differences in the correlations of learning between urban and rural older adult groups,^1^ the raw sample data were processed for case selection using SPSS, and correlation analysis was conducted for both separately (Table 7). Data analysis revealed a significant positive correlation (*p* < 0.01) between older adult learning (learning investment, learning atmosphere, and learning experience) and SWB (including physical and mental health experience, self-development experience, and adaptation and satisfaction experience) of urban and rural older adults. Learning investment had a low correlation with older adults’ SWB (*r* = 0.139), learning atmosphere had a moderate correlation with SWB (*r* = 0.774), learning experience had a strong correlation with SWB (*r* = 0.851), and the latter two were more closely related to older learners SWB. There was a positive correlation between senior learning and learners’ SWB. Accordingly, Hypothesis 1 was well verified.

**Table 7:** Correlation between variables.

		Learning input	Learning atmosphere	Learning experience	Physical and mental health experience	Self-development experience	Adaptation to meet experience	Subjective well-being
Learning input	Rural area	1						
	City							
Learning atmosphere	Rural area	0.103^**^	1					
	City	0.119^**^						
Learning experience	Rural area	0.090^*^	0.817^**^	1				
	City	0.143^**^	0.842^**^					
Physical and mental health experience	Rural area	0.110^**^	0.727^**^	0.831^**^	1			
	City	0.117^**^	0.724^**^	0.795^**^				
Self-development experience	Rural area	0.136^**^	0.729^**^	0.836^**^	0.930^**^	1		
	City	0.149^**^	0.751^**^	0.834^**^	0.916^**^			
Adaptation to meet experience	Rural area	0.142^**^	0.756^**^	0.846^**^	0.905^**^	0.924^**^	1	
	City	0.138^**^	0.777^**^	0.846^**^	0.887^**^	0.919^**^		
Subjective well-being	Rural area	0.132^**^	0.757^**^	0.860^**^	0.973^**^	0.979^**^	0.966^**^	1
	City	0.139^**^	0.774^**^	0.851^**^	0.967^**^	0.976^**^	0.963^**^	

* *p* < 0.05;

** *p* < 0.01;

^***^p < 0.001.

### Independent *t*-test of Urban–Rural Differences in Subjective Well-Being of Older Learners

To further clarify the differences between the SWB of urban and rural older adult learners, independent *t*-test was performed on the sample data (Table 8). Results showed significant differences between rural and urban older learners in terms of learning investment, learning atmosphere, and learning experience. Learning atmosphere and learning experience had higher scores in rural than in urban older learners, while the opposite was true for learning investment. In addition, there were significant differences in physical and mental health experience, self-development experience, and adaptation satisfaction experience. In terms of mean values, physical and mental health experience, self-development experience, and adaptation satisfaction experience were significantly stronger in rural older learners than in urban older learners. Overall, SWB differed between urban and rural older learners. Therefore, Hypothesis 2 was supported.

**Table 8:** Results of independent-samples *t*-test (*n* = 2007).

Variables		Rural	Cities and towns	*t*	*p*	Cohens’ *d*
		*M*(*SD*)	*M*(*SD*)			
Older adult Learning	Learning investment	3.65(0.596)	3.9(0.649)	−8.472^***^	0.000	−0.4
	Learning atmosphere	5.6(0.694)	5.45(0.791)	4.557^***^	0.000	0.2
	Learning Experience	5.63(0.637)	5.44(0.824)	5.27^***^	0.000	0.25
SWB	Physical and mental health experience	5.58(0.752)	5.38(0.911)	5.086^***^	0.000	0.24
	Self-development experience	5.61(0.752)	5.42(0.881)	4.743^***^	0.000	0.23
	Adapt to meet the experience	5.67(0.654)	5.51(0.79875)	4.806^***^	0.000	0.22

*** *p* < 0.001.

### Multiple Regression Analysis

The results of interaction effect analysis (Table 9) show that there was a significant effect of older adult learning on SWB (*F* = 32.189, *p* = 0.000 < 0.05, *η*^2^ = 0.745); there was a significant effect of type of household registration on older adults SWB (*F* = 8.036, *p* = 0.005 < 0.05, *η*^2^ = 0.005); the interaction of senior learning and type of household registration had a significant effect on SWB (*F* = 1.513, *p* = 0.002 < 0.05, *η*^2^ = 0.073). Therefore, regression analysis was conducted with different samples from rural and urban areas to discern the urban–rural variability of the effect of learning factors on the SWB of older learners.

**Table 9:** Interaction effect analysis.

	*df*	Mean	*F*	*p*	*η^2^*
Senior Learning	159	5.326	32.189	0	0.745
Household registration	1	1.33	8.036	0.005	0.005
Senior Learning **×**Household registration	91	0.25	1.513	0.002	0.073

Given the numerical characteristics of the study variables, multiple linear regression analysis with ordinary least squares was used to estimate the parameters of the factors influencing SWB of older learners. The study used age, gender, education, marital status, and monthly income as control variables, SWB as the dependent variable, and learning investment, learning atmosphere, and learning experience as independent variables. *Y* was the dependent variable (SWB), *X_1_,X_2_...X_k_* were the independent variables (older learners and their factors), and the multiple linear regression model was set as follows (Table 10):


(1) Yi=β0+β1X1+β2X3…+βiXi+ε

**Table 10:** Regression model for the subjective well-being of urban and rural older adults.

Variables	Subjective well-being (full sample)				Subjective well-being (rural subsample)				Subjective well-being (urban subsample)			
	*B*	*SE*	*β*	Sig	*B*	*SE*	*β*	Sig	*B*	*SE*	*β*	Sig
Learning investment	0.030^*^	0.014	0.025	0.031	0.059^**^	0.021	0.051	0.005	0.023	0.019	0.018	0.237
Learning atmosphere	0.192^***^	0.022	0.184	0	0.159^***^	0.031	0.158	0	0.208^***^	0.029	0.197	0
Learning Experience	0.714^***^	0.021	0.699	0	0.756^***^	0.032	0.727	0	0.692^***^	0.028	0.682	0
*R* ^2^	0.743				0.751				0.735			
*F*	1926.256^***^ (sig = 0.000)				774.831^***^ (sig = 0.000)				1138.128^***^(sig = 0.000)			

* *p* < 0.05;

** *p* < 0.01;

*** *p* < 0.001.

Regression analysis of the overall sample of older learners revealed that the standardized equations for the three multiple linear regressions for the full sample, the rural subsample, and the urban subsample were:


$$\begin{array}{c} Y\;\left(\mathrm{full sample}\right)=0.025\times \mathrm{learning input}\\ +0.699\times \mathrm{learning experience}\\ +0.184\times \mathrm{learning atmosphere} \end{array}$$

$$\begin{array}{c} Y\;\left(\mathrm{rural subsample}\right)=0.051\times \mathrm{learning engagement}\\ +0.772\times \mathrm{learning experience}\\ +0.158\times \mathrm{learning climate} \end{array}$$

$$\begin{array}{c} Y\;\left(\mathrm{town score sample}\right)=0.682\times \mathrm{learning experience}\\ +0.197\times \mathrm{learning atmosphere} \end{array}$$

Combining Table 6 and the standard equation of the regression model, it can be seen that learning engagement, learning atmosphere, and learning experience have a positive predictive effect on the SWB of older learners (*F* = 1926.256, *p* < 0.001, *R^2^* = 0.743), which can explain 74.3% of the variance probability of SWB, among which learning engagement has a weak effect on the SWB of older learners. The regression results of SWB of rural older learners showed that learning engagement, learning atmosphere, and learning experience had significant positive effects on SWB (*F* = 774.831, *p* < 0.001, *R^2^* = 0.751), which could explain 75.1% of the variance in SWB. The regression results of SWB of urban older learners showed a significant positive effect of learning atmosphere and learning experience (*F* = 1138.128, *p* < 0.001, *R^2^* = 0.735), which could explain 73.5% of the variation in SWB, but learning engagement (*p* = 0.237 > 0.05) had no corresponding effect.

The comparison of urban and rural older adult learners shows that learning commitment, learning atmosphere, and learning experience all have a significant positive effect on the SWB of rural older adult learners and better explain their SWB, while only learning atmosphere and learning experience have a positive predictive effect on the SWB of urban older adult learners and more poorly explain SWB of this group. In other words, there were some urban–rural differences in senior learning’s effect on participants’ SWB. Therefore, Hypothesis 3 was well verified.

## Discussion

The purpose of this study was to explore the differences between the SWB of older learners from urban and rural areas in China. A questionnaire was used to examine urban–rural differences in SWB by randomly distributing electronic questionnaires at educational institutions, such as senior universities, community colleges, and adult schools. The results found that (1) Older adult learning can effectively improve the SWB of urban and rural seniors; (2) there is a significant difference between the SWB of urban and rural older learners, and rural older adults have a higher level of SWB than their urban counterparts; and (3) there is an urban–rural difference in learning participation’s effect of on older learners’ SWB.

Older adult learning contributes to the SWB of urban and rural senior learners, i.e., learning engagement, learning atmosphere, and learning experience all play a positive role in influencing their SWB. These results align with previous studies. For example, Klimova et al. (2021) showed that older adults’ overall satisfaction and SWB were very high when they participated in foreign-language courses. Bo (2017) also showed that participation in learning activities was significantly and positively associated with SWB.

There was a significant difference between urban and rural older learners’ SWB, with rural older learners having better SWB than their urban counterparts. Similar findings have been obtained in other studies. Educational activities can improve people’s outlook perceptions, sense of self-control, and growth potential, which in turn can help enhance rural farmers’ SWB (Jin and He, 2012). Social comparison theory suggests that people’s experience of happiness is usually related to the process of comparing things or behaviors; by comparing with others, they obtain the corresponding subjective happiness response (Cui, 2014). In this research, we found that rural older learners’ learning environment is in a more homogeneous learning state, individuals do not have high expectations of learning, and their SWB will be significantly increased with continuous participation in learning. In contrast, the learning environment and individual needs of urban older learners tend to be higher, which reduces their SWB scores to a certain extent.

Similar studies have demonstrated that the following factors also affect the SWB of older learners (Wu and Chen, 2014; Bo, 2017; Su and Zhang, 2021). Researchers have found that social interaction plays an important role in older adults’ psychological well-being and quality of life (Hupkens et al., 2018; Soares et al., 2021; Lee et al., 2022). Accordingly, participation in education for older adults is a beneficial social interaction with others and can contribute to improving older adults’ SWB, which is in accordance with Hypotheses 1 and 3.

The results of this study show that learning investment, learning atmosphere, and learning experience all facilitate rural older learners’ SWB, and only learning atmosphere and learning experience facilitate urban older learners’ SWB, so there is an urban–rural difference in the impact of older learning. The reason learning inputs more significantly affect rural older adults’ SWB is that the inputs, including behavioral, emotional, cognitive, and monetary inputs, in the process of participating in learning and education activities lead older adults to gradually recognize the value of the learning content, stimulate their potential, form positive emotional experiences, and enhance SWB (Sun and Liu, 2020). As for urban older adults, who have higher educational attainment than rural older adults, education raises subjective expectations to a certain extent, thus negatively affecting SWB, which confirms previous research. Stutzer (2004) found that people who received secondary education generally had higher well-being than those who received lower and higher education, and life expectations were more likely to be met.

Finally, given the importance of close relationships for older adult learning and SWB, more efforts should be made to address the inequalities. This paper proposes the following strategies for learning activities of urban and rural older adult learners and the improvement of their SWB: (1) Renew the concept of education, adhere to the overall planning of urban and rural areas, and promote equality of education in urban and rural areas for older adults; (2) Make the supply of educational resources precise, focusing on improving learning effectiveness for older adults; and (3) Enhance older adults’ emotional experience by creating a good learning atmosphere. These findings have significant implications for the development of successful aging, and may offer useful guidance for professional counseling for Chinese older adult learners.

### Limitations and Future Directions

Some limitations of this study should be noted. First, the survey data came from the middle and eastern regions of Zhejiang, Anhui, and Shandong Provinces, where the development of senior education is enhanced, with increased representation; however, the sample may not be representative of the population nationally, which limits the scope of promotion. Second, due to the cross-sectional design, the causal relationship of main study variables should be further confirmed in the future studies. Third, the use of the Internet and institutional social media may have caused a bias against older adults who cannot use the Internet. Finally, although we pointed out that there are differences in the effects of older adult learning on urban and rural older learners’ SWB, there are many other educational factors, such as education gap, that can cause differences, which will be our focus in future studies.

Without disregarding these limitations, our results introduce diverse policy implications aimed at supporting older adults in their learning to contribute to their SWB. On the one hand, echoing China’s policy reports, our findings posit the urgency of generating strategies to assist older adult education agencies and older adult learners in strengthening SWB. On the other hand, our study explores the urban–rural differences in SWB and the influencing mechanisms of urban–rural older adults in terms of learning participation, which is unique to our study. While previous studies have focused on the influence of objective factors on older adults’ SWB, this study provides a new perspective to study the differences between older adults. Additionally, our study has a large sample size to be of practical guidance. Finally, through empirical research, this study truly reflects the current situation of older adults’ participation in senior education, objectively measures their SWB, comprehensively analyzes the relationship between senior education and older adults’ SWB, and enriches the relevant theoretical research.

## Data Availability Statement

The original contributions presented in the study are included in the article/supplementary material, further inquiries can be directed to the corresponding authors.

## Author Contributions

All authors listed have made a substantial, direct, and intellectual contribution to the work and approved it for publication.

## Funding

This study was funded by the special subject “Research on the Construction of a Service Guarantee System for Elderly Education” of Zhejiang Provincial Social Science Planning “Research on the Spirit of the Fifth Plenary Session of the 19th Central Committee of the Communist Party of China” (no. 21WZQH16YB), and the 2019 General Project of Education “Happiness Measurement and Empirical Research on the Elderly from the Perspective of Education Premium” (no. BKA180235) of the National Social Science Foundation of China.

## Conflict of Interest

The authors declare that the research was conducted in the absence of any commercial or financial relationships that could be construed as potential conflicts of interest.

## Publisher’s Note

All claims expressed in this article are solely those of the authors and do not necessarily represent those of their affiliated organizations, or those of the publisher, the editors and the reviewers. Any product that may be evaluated in this article, or claim that may be made by its manufacturer, is not guaranteed or endorsed by the publisher.

## Footnotes

^1^Note: The division of urban and rural elderly learners is based on the survey respondents’ household registration type.
